# Self-Organized Proactive Routing Protocol for Non-Uniformly Deployed Underwater Networks

**DOI:** 10.3390/s19245487

**Published:** 2019-12-12

**Authors:** Waheeduddin Hyder, Miguel-Ángel Luque-Nieto, Javier Poncela, Pablo Otero

**Affiliations:** 1 Department of Ingeniería de Comunicaciones, University of Málaga, 29010 Málaga, Spain; waheed@uma.es (W.H.); jponcela@uma.es (J.P.); pablo.otero@uma.es (P.O.); 2 Department of Computer Science, Faculty of Science and Technology, Ilma University, Karachi 75190, Pakistan

**Keywords:** underwater, UWSN, location free, routing protocol, self-organized, self-configud, wireless networks, proactive

## Abstract

Electromagnetic (EM) waves cannot propagate more than few meters in sea water due to the high absorption rate. Acoustic waves are more suitable for underwater communication, but they travel very slowly compared to EM waves. The typical speed of acoustic waves in water is 1500 m/s, whereas speed of EM waves in air is approximately 3 × 10^8^ m/s. Therefore, the terrestrial wireless sensor network (WSN) protocols assume that the propagation delay is negligible. Hence, reactive protocols are deemed acceptable for WSNs. Other important issues related to underwater wireless sensor networks (UWSNs) are determining the position of the underwater nodes and keeping a time synchronization among the nodes. Underwater nodes can neither determine their position nor synchronize using Global Navigation Satellite Systems (GNSS) because of the short penetration of EM waves in sea water. The limited mobility of UWSN nodes and variation in the propagation speed of acoustic waves make time synchronization a challenging task for underwater acoustic networks (UASNs). For all these reasons, WSN protocols cannot be readily used in UASNs. In this work, a protocol named SPRINT is designed to achieve high data throughput and low energy operation in the nodes. There is a tradeoff between the throughput and the energy consumption in the wireless networks. Longer links mean higher energy consumption. On the other hand, the number of relay nodes or hops between the source node and the final destination node is a key factor which affects the throughput. Each hop increases the delay in the packet forwarding and, as a result, decreases the throughput. Hence, energy consumption requires the nearest nodes to be chosen as forwarding nodes, whereas the throughput requires the farthest node to be selected to minimize the number of hops. SPRINT is a cross-layer, self-organized, proactive protocol which does not require positioning equipment to determine the location of the node. The routing path from the node to the gateway is formed based on the distance. The data sending node prefers to choose the neighbor node which is closest to it. The distance is measured by the signal strength between the two nodes.

## 1. Introduction

**Figure 1:**
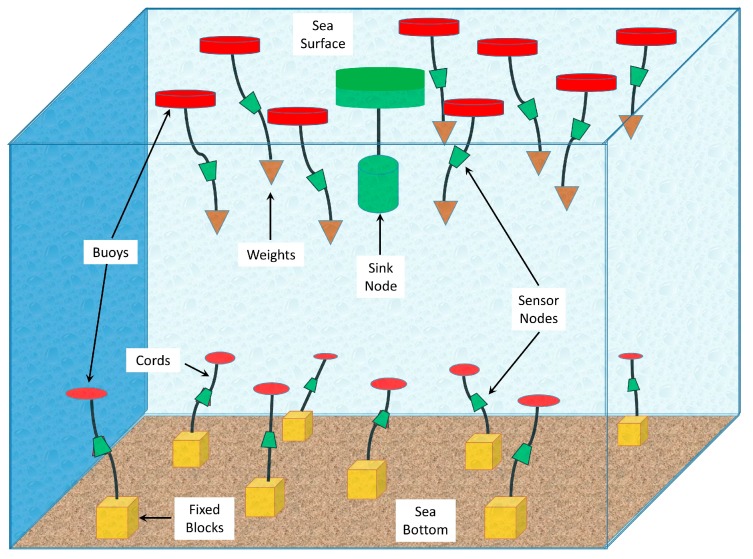
3D underwater wireless sensor networks (UWSN) architecture.

Underwater wireless sensor networks (UWSNs) have many applications related to environmental monitoring, disaster alerts, and military surveillance. UWSN may be deployed in either shallow or deep waters. When a sensor network is deployed in shallow waters and sensors are attached to the bottom of the sea, a 2D deployment of the network is considered. In deep waters, the nodes are suspended by a rope or a chain, which is attached to the surface buoys or the anchors at the bottom of the sea (Figure 1). The suspended sensor nodes in 3D networks keep moving in all directions. This movement is limited by the length of the connecting rope or chain. The continuous random movement of the nodes makes the protocols design more challenging in 3D networks.

**Figure 2:**
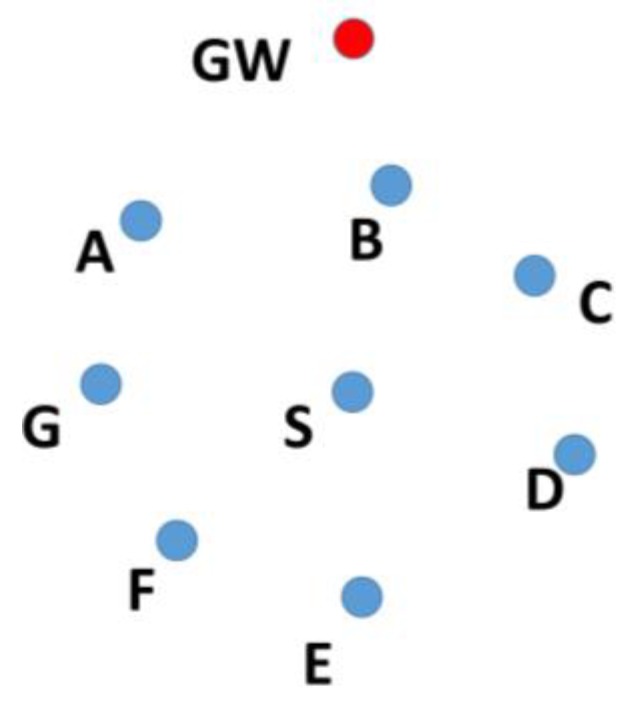
Selection of relay nodes.

In a cooperative network, the nodes farther away from the sink node, also known as the gateway (GW) node, send their data to the GW with the help of intermediate nodes known as relay nodes. To select a relay node, the position of its candidate nodes should be known. For example, nodes A, B, C, D, E, F, and G in Figure 2 are at one hop distance from node S. All of these nodes will receive the data transmitted by node S, but only the nodes closer to the GW should retransmit the data. Therefore, before node S can select one of these nodes, it needs to know the position of nodes A, B, C, D, E, F, and G.

In WSNs, determining the location of a sensor node is trivial. The sensor nodes can directly determine their position with the help of Global Navigation Satellite Systems (GNSS). However, this process in UWSNs is a challenging task because the GNSS signal is not present. One way to determine the position of the underwater nodes is that the GW gets its position with help of GNSS and the rest of the nodes determine their position with respect to the GW. However, in a 3D network, it is very difficult to determine their position by this method because of the continuous limited movement of the nodes.

In UWSNs, the position of the nodes can also be determined by Time Difference of Arrival (TDoA) [1] and Time of Arrival (ToA) [1]. TDoA is based on the two different transmission-media, like radio frequency and acoustic wave. The distance is estimated by different arrival times due to the dissimilar velocities of the radio wave and acoustic wave [2]. However, EM waves are not available and TDoA cannot be used in underwater environments. The time of Arrival (ToA) technique is an alternative approach; it is based on the travel time of the acoustic wave from the source to the destination. The sender stamps the time of sending the packet and the receiver calculates the travel time by comparison to its local time and estimates the distance. This method requires time synchronization between the sender and the receiver nodes. Achieving time synchronization among the nodes in a UWSN is very difficult because of variance in the acoustic waves’ propagation speed and limited mobility of the sensor nodes caused by the water currents.

As an alternative method, the position of the nodes can be determined by measuring their distance from the water surface by means of depth sensors. This method is used by many routing protocols, like DBR [3], LB-AGR [4], and VBF [5]. However, depth sensors have their own disadvantages, like measurement errors, increase in power consumption due to depth sensing and computation, and the extra cost [6].

Because of the constraints and disadvantages mentioned above, the routing protocols which are based on neither the location of the sensor nodes or use the depth sensors are more practical and beneficial. In this paper, we use the distance between the sender node and the candidate nodes as a metric of selection for the relay node, estimated by the received signal strength (RSS).

After a review of the state of the art of underwater routing protocols in next section, the three metrics used in the new protocol proposed are explained in detail (Section 3). Later on, the process to build the routes is examined (Section 4) and a mathematical analysis is performed (Section 5). Next, a suitable packet format is stablished in Section 6, and results of simulations are presented (Section 7) with a discussion of their importance. Finally, the final conclusions are remarked.

## 2. Related Work

### 2.1. Protocols Based on RSS

The reliable energy-efficient cross-layer routing protocol (RECRP) [7] uses RSS to estimate the distance between the nodes and choose the shortest path. RECRP selects the forwarding node based on distance, residual energy of the sender, and the potential forwarding node and hop count to the sink node. It is also assumed that the nodes are time synchronized. Once the routing update phase is over, the path information is regularly updated using data packets. To update the routing path information regularly, the data packets contain residual energy transmission power information. The neighbor nodes always update their routing table when they receive the data packet, even though they are not the relay node. This increases the data packet overhead cost and unnecessary processing, which increases the energy consumption.

In [8], the authors propose a routing protocol which uses the RSS to adapt the transmission power to conserve the node energy. The master node initiates the network configuration process by transmitting a topology discovery message (TDM). The nodes will propagate the packet through all the nodes in the network. This way, each node will make a list of all of its neighbors and forward that list to the gateway to select the most appropriate path for each node. The master node selects the path based on the available capacity across the path and number of hops. This protocol has some serious problems. Firstly, it has centralized the path selection process. All the nodes collect the data and send them back to the gateway node, which establishes a routing path for the nodes. If any node goes down, then the master node will not be able to inform the other nodes of the status before the next periodic transmission of TDM. In order to keep the master node more accurately updated with changes in the topology, the periodic transmission of TDM must be quite frequent. In [1], the RSS measurement for short-distance (0–1000 m) was used for underwater acoustic communication and, once again, the distance measurement was used to determine the location of the nodes. The Lambert W function, also known as product log or omega function, calculates the distance using RSS. Lambert W functions are evaluated by Taylor series or numerical methods like the Newton or Halley methods (this last one was used in [1] to approximate distance in four iterations). However, fast convergence using the Halley method increases computational complexity and requirements in the power supply to the processor, which means a larger delay and energy consumption.

### 2.2. Location-Based Protocols

There are other routing protocols for UWSNs which are based on the depth of the sensor nodes, like LB-AGR, VBF, and DBR. The packet forwarding mechanism in LB-AGR is based on the level-difference between the neighbor nodes, available power, node density, and location. The traffic is routed according to its type as well. The traffic type is mainly divided into two categories: upstream and downstream. The traffic from the sensor nodes to the sink through is called the upstream traffic, and the opposite way is downstream traffic. The downstream traffic is further divided into three categories. The first is the downstream traffic for a designated area, the second is the downstream for a specific node, and the third is downstream for all the sensor nodes.

Vector-based forwarding (VBF) is a location-based protocol based on the concept of virtual pipes. Nodes are selected from the source to the sink, which can possibly forward the packets. These selected nodes form a shape like a pipe, hence it is called the “virtual pipe”. To avoid the issues of packet losses and node failures, multiple nodes within the virtual pipe forward the data packets. One major drawback is that the isolated nodes may create voids in the low-density network. The power consumption is also high due to three-way handshaking mechanism. Hop-by-hop VBF (HH-VBF) [9] proposes a virtual pipe node that is nodes-based, rather than from source to destination, to overcome the voids issue in VBF. Since each node forms a new virtual path before forwarding the packet, the voids are eliminated. HH-VBF uses more control packets to form a virtual pipe for each forwarding node than VBF, so the average energy consumption is poor due to broadcasting, but the packet delivery ratio is better than LB-AGR.

Depth-based routing (DBR) is based on greedy routing. The path to the gateway is chosen depending on the depth of the next hop. Each forwarding node sends its depth information to the next hop in order for all the nodes to know about the depth of their neighbor nodes. The node which has the lowest pressure, or in other words, is closest to the gateway, will be selected as the next forwarding node. In the energy-efficient depth-based routing (EEDBR) [10] protocol, the nodes share their depth and residual energy information with their neighbors. Those with high residual energy are most likely to forward the packet, and those with low energy will try to avoid forwarding. There is a threshold value for residual energy to choose the node with higher residual energy: if the node has a residual energy higher than this threshold value, it will forward the packet.

HydroCast [11] is similar to DBR but it avoids forwarding the packets to such a node which is not closer to the destination than the current node using the NADV link metric [12]. First, the nodes with higher priority will forward the packet. If they fail, then the nodes with lower priority will try. To avoid collision and redundant packets, the nodes with the lower priority will refrain from transmitting the packet and will listen to the packet forwarding by the highest priority nodes. This is possible because they are within the transmission range of each other.

Like DBR and HydroCast, void-aware pressure routing (VAPR) [13] also uses the depth information, hop count, and sequence number to find the next hop and path to the sink. Sink nodes initiate the configuration process by transmitting beacon packets. A node decides its forwarding direction after receiving the beacon message from the deeper node or shallower node. The forwarding direction is set towards the sea surface if the beacon is received from a shallower depth node and towards the sea bottom if received from a deeper node. This way, the voids can be avoided by a change in packet forwarding direction. The energy-efficient routing protocol (EUROP) [14] is another depth-based protocol which uses pressure sensors. The depth of the nodes can be changed by an inflating device which creates multiple layers of nodes from the bottom of the sea to the ocean surface.

### 2.3. Opportunistic Protocols

Energy-efficient opportunistic routing technology (EFFORT) [15] uses opportunistic end-to-end cost (OEC) to reduce the number of candidate nodes from source to destination. This way, the network lifetime increases. The OEC cost is determined based on a candidate node’s residual energy and link reliability.

Energy-efficient and obstacle-avoiding routing (EOAR) [16] uses fuzzy logic to select the forwarding node. The selection is made based on residual energy, the angle between the two nodes, and the propagation delay. Packets are forwarded based on priority to avoid the collisions. The propagation delay is calculated as the time difference between the local current time of the node and the time-stamp included in the received packet. This method requires time synchronization between the nodes, which is a challenging task for UWSNs due to the varying propagation delay and some degree of mobility.

GDGOR-IA and GRMC-SM [17] use the location information of the nodes to form the routing path, which is established when the data are to be forwarded, thus increasing the routing delay. The data forwarding uses the greedy approach. The network is divided in logical cubes and a set of nodes is selected in each cube to forward the data packet. PICS and PRCS [18] use depth information and ToA to calculate the position of the sender and distance between the nodes. PICS uses two hops information to select the routing path, whereas PRCS uses only one hop information. In order to select the candidate nodes to forward packets, a new metric named EEPA [18] (enhanced expected packet advance) is applied, which considers the channel quality and path correlation between two neighbors.

The shortcomings of the protocols discussed above are listed as below:

1. In location-based routing protocols:
  - The sensor nodes determine their position with respect to the GW, which in turn determines its position via GNSS. Due to the varying speed of acoustic waves and the mobility of the nodes, the probability of error in position determination is quite high;
  - The position determination by the depth sensors increases the power consumption and cost of the sensor nodes;
2. Time synchronization is required;
3. Additional devices like a depth sensor and array of antennas (for the angle of arrival method [1]) are required;
4. Centralized routing path formation control;
5. The required computational processing delay and power are high;
6. The routing protocols are reactive.

In this paper, we designed a routing protocol which addresses all these shortcomings. Our proposed proactive protocol requires no location information, no time synchronization, and no additional devices, and uses less computational power.

## 3. Overview of the SPRINT Protocol

The RSS metric is used to estimate which node is the closest neighbor of the transmitting node. The node selects the node that is closest to it to forward the packet. Along with the distance, two more metrics are used: number of hops between the sender and the sink, and the number of neighbors of the candidate nodes. The minimum distance between the nodes also increases the probability of successful packet delivery.

**Figure 3:**
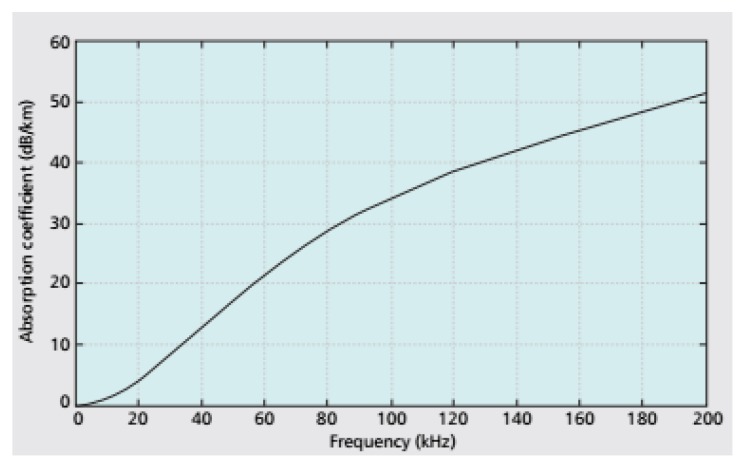
Absorption coefficient versus frequency.

The estimation of the RSS is not very precise due to the path loss, the fading, and the limited mobility of the sensor nodes. The spreading of acoustic signal is an important factor for path loss. Spreading losses depend not only on the transmission range, but also on the propagation model adopted: cylindrical (shallow waters) or spherical (deep waters). The path loss is also due to absorption. The absorption of acoustic channels depends on the frequency of the acoustic signal. Total path loss A(l,f) [19] due to spreading and absorption can be expressed in decibels by the following equation:

(1) $$A\left(l,f\right)=10\cdot \log\left(l^{k}\right)+\alpha \left(f\right)\times l\;(\mathrm{dB}),$$

where l (km) is the transmission range of the acoustic signal, k is the spreading factor, α(f) (in dB/km) is the absorption coefficient, and f (kHz) is the frequency. The value of k=1 is for cylindrical spreading, and k=2 for spherical spreading. A graph of absorption coefficient versus acoustic signal frequency is depicted in Figure 3 [20]. We can see that for frequencies up to 20 kHz, α(f) is <4 dB/km (approximately).

**Figure 4:**
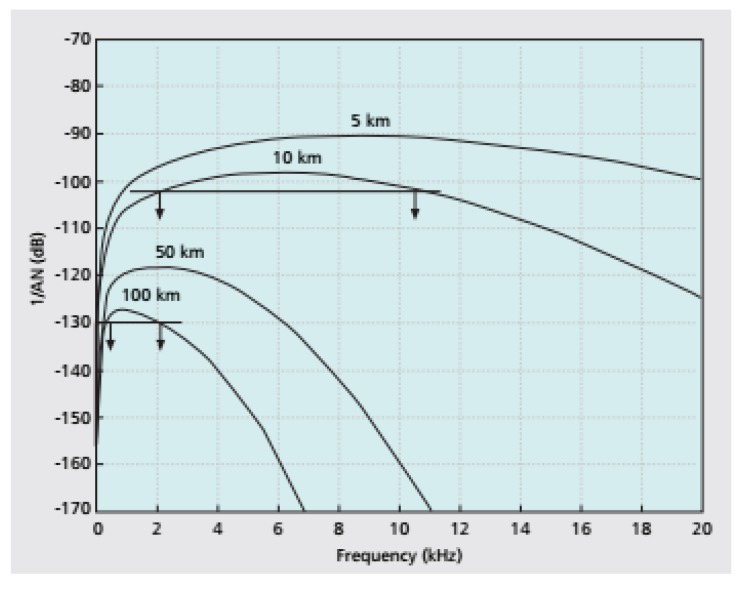
SNR versus frequency and distance.

Figure 4 [20] shows that Signal-to-noise ratio (SNR) also depends on the frequency. This figure also shows that the bandwidth of acoustic signal depends on the distance. For 100 km, the bandwidth is about 1 kHz, whereas for 5 km it is about 10 kHz.

Fading is more severe in shallow water compared to deep water due to multipath. There are no standard fading models for acoustic communication, and experimental measurements are used to predict the channel behavior [20]. For this protocol, the error in estimation of RSS due to losses, fading, and limited mobility of the nodes is not significant as long as a node successfully forms a path with one of its neighbors that is closer to the gateway. The error in node selection may be corrected later by measuring the RSS value at regular intervals using the data packets.

Our proposed protocol assumes that the nodes are deployed randomly in a 3D network. The minimum distance between nodes is 300 m and maximum is 1000 m. The transmission power is adaptable and can be adjusted according to the distance between the transmitter and sender. The nodes are not stationary and keep moving in all directions, although their movement is limited by the length of the binding lines to the surface buoy or the anchor at the sea bottom (see Figure 1). Lists of the acronyms and symbols used in this paper are shown in Table 1 and Table 2, respectively.

**Figure 5:**
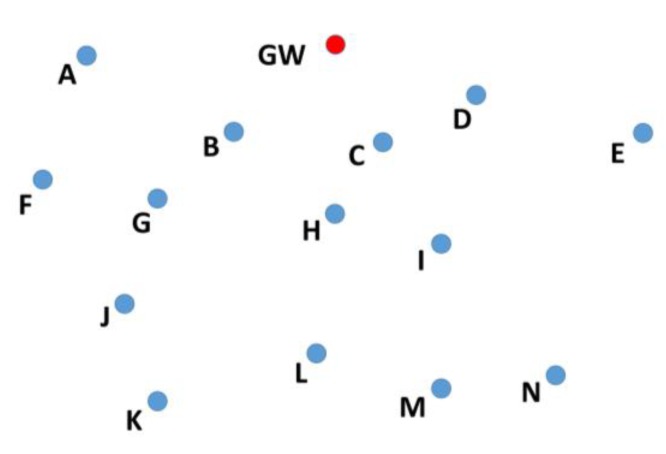
Non-uniformly deployed nodes in 2D.

The process of route formation is initiated by the gateway node. The gateway broadcasts a route request (RR) packet, which will traverse throughout the network. When a node receives the RR packet, it measures the strength of the received signal. Initially, all the nodes will transmit at the same power, which will be enough to cover the maximum distance between the nodes. With the help of RSS, the distance between the RR sending node and receiving node is estimated. The RSS indicates the relative proximity between the sending node the receiving node. This is explained in the following example. Assume that three nodes, B, C, and H, are placed as illustrated in Figure 5.

Suppose that nodes B and C have received the RR packet from the gateway. Nodes B and C will broadcast the RR packet at randomly selected time slots. Since H is the neighbor of both nodes, it will receive the RR packets from nodes B and C. When H receives the RR packet from B, it measures the RSS value and records it in a table. Similarly, H records the RSS value when it receives the RR packet from C. After that, it will compare the RSS value of both B and C, and will choose the node which has the stronger RSS. In this case, H will choose C as the next hop to forward the packet towards the gateway, because it has stronger RSS compare to B. The H node chooses the node having stronger RSS because it assumes that strong RSS is due to the shorter distance. However, before making the final decision, H will consider both RSS values, the number of hops between the candidate nodes and the gateway, and the number of neighbors of the candidate nodes.

**Figure 6:**
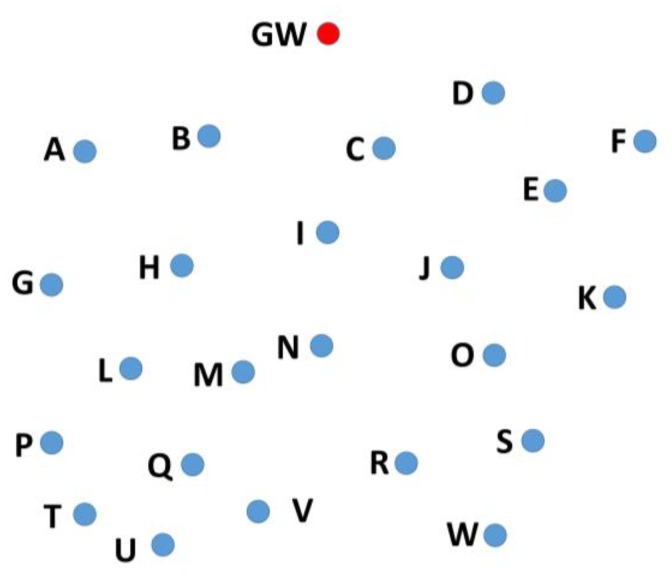
Example of three metrics-distance, neighbors, and hops.

In the case of a short distance between the sender and the receiver, less transmission energy is required by the sender to transmit the packet. Therefore, when the weight of the RSS is set to maximum (i.e., 1), the closer candidate node is selected. This saves energy at the sender node. However, this approach may decrease the throughput if too many hops are added in the path between the original packet sender and the gateway. Therefore, two more metrics are considered at the time of selection of the forwarding node: number of hops and number of neighbors. The first one is because the number of hops affects the energy consumption and throughput. The second one is because a high number of neighbors means a better chance to be selected as the forward node, so it is a good metric to be considered. To better understand the idea, a scenario is considered. Suppose that a node, V, has two candidate nodes, Q and M, as shown in Figure 6. Node Q is 300 m away from node V, has five neighbors (U, T, P, L, M) and can reach the gateway in four hops (L–H–B–GW), whereas node M is 600 m away from node V, has four neighbors (Q, L, H, N) and can reach the gateway in three hops (H–B–GW). At the time of forwarding node selection, node V will select the node based on all three metrics. The weights given to each metric will influence the selection accordingly. The selection is based on these three metrics to achieve a good balance between the throughput and the energy consumption. Suppose the shortest distance is given the highest weight, then node Q will be selected. If number of neighbors is given the highest weight, then node M will be selected, and if number of hops is given the highest weight then again M will be preferred over Q.

The values of the metrics will be normalized and multiplied by their weights (from 0 to 1). Prior to the deployment of the nodes, the weights will be assigned for each metric of selection.

**Figure 7:**
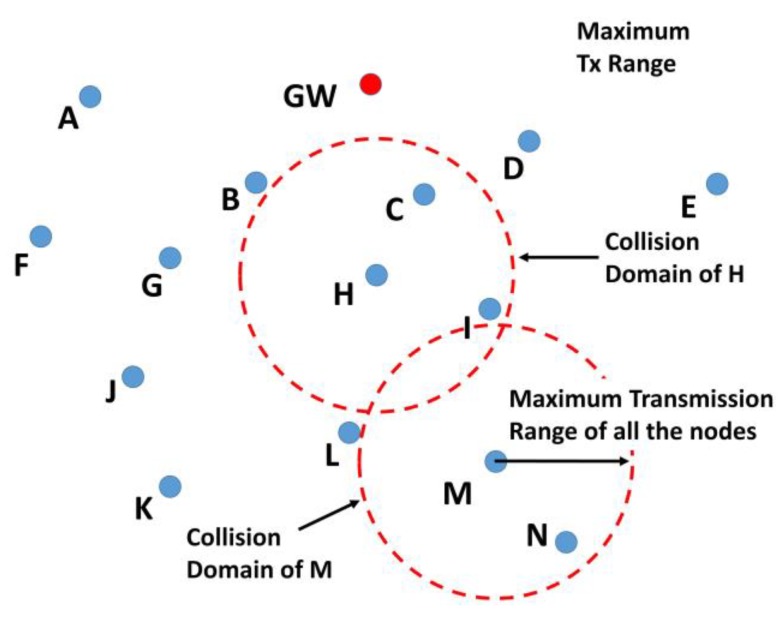
A typical collision domain.

Packet collision at the receiver causes packet loss. The collision occurs when two or more nodes send packets to a node such that the packets arrive at the receiving node overlapped in time. All the nodes which can cause a collision in their transmission range form a collision domain, as shown in Figure 7. A network may have multiple collision domains. The number of nodes in a collision domain depends on the node’s density and their transmission range. In order to avoid a collision, the nodes will send the RR packet at a randomly selected time slot from a set of possible time slots. Twenty time slots in the set are assumed to be sufficient for a moderately dense network. However, the number of slots may be increased in a densely populated network. The interval between two consecutive time slots will comprise packet delay, propagation delay, and guard time. For the latter, we assume that 5% of the total of packet delay and propagation delay will be enough to adjust the variance of the end to end packet transmission delay due to variations in the acoustic propagation velocity. When a node sends the RR packet, it will wait for the Route Request Acknowledgement (RR_ACK) packet from at least one node.

Once the RR packet has gone through all the nodes in the network, the nodes at end of the network send a Route Request Response RR_RSP packet back to the gateway. Nodes assume they are end nodes when they do not receive any response packet after sending the RR packet. When the gateway receives the RR_RSP packet from all the first hop nodes it assumes that all the nodes have successfully formed the path to the gateway. The nodes keep measuring the RSS of the received packets during the data packet transmission to optimize the path. If a node finds a closer neighbor compared to the existing forwarding node, then the former selects the latter as the next hop to forward the packets.

## 4. Route Formation Process

The route formation process starts with broadcasting the RR packet from the gateway, which is forwarded through all the network nodes. The steps for broadcasting the RR packet are described below.

### 4.1. Forwarding the RR Packet

1. GW broadcasts an RR packet three times with its node ID to ensure that all the neighbor nodes have received it;
2. The nodes that receive the RR packet form the path with the GW without considering RSS, because they are at one hop distance from the GW;
3. The nodes that receive the RR packet broadcast an RR packet at randomly selected time slots to avoid collision;
4. A node might receive the RR packet from only one node or multiple nodes:
  4.1. If it receives the RR packet from just one node, then it forms its path to the GW through that node;
  4.2. If it receives the RR from multiple nodes, then it compares the RSS values, hop counts to the GW, and number of neighbors of the candidate nodes. It will select the forwarding node according to the weights given to RSS, the least hop count, and least number of neighbors to form the path;
5. Once the forwarding node is selected, the acknowledgement of the RR packet (RR_ACK) is sent. An RR sending node receives acknowledgment from all nodes which are within its transmission range;
6. Steps 1–5 (broadcast of RR packet) are repeated by all the nodes that received the RR packet earlier, until all the nodes in the network have received the RR packet.

The RR packet is transmitted, at the most, three times, if acknowledgement is not received. If that occurs, then the node concludes that it is an end node and it will start sending a response (RR_RSP) packet that contains its own identification number and the identification numbers of the forwarding nodes. The RR_RSP packet is not broadcast, but sent to the node which was earlier selected by each node as a packet forwarding node.

### 4.2. Selection Criteria

The forwarding node can be selected based on RSS, number of hops, and the number of neighbors of the forwarding node. If the protocol is used for delay sensitive data, then the smaller number of hops will be given more weight. If energy conservation is the prime goal, then RSS and least number of neighbors will be given preference over the number of hops. Below, a scenario is considered to understand the process of selection in more detail.

**Figure 8:**
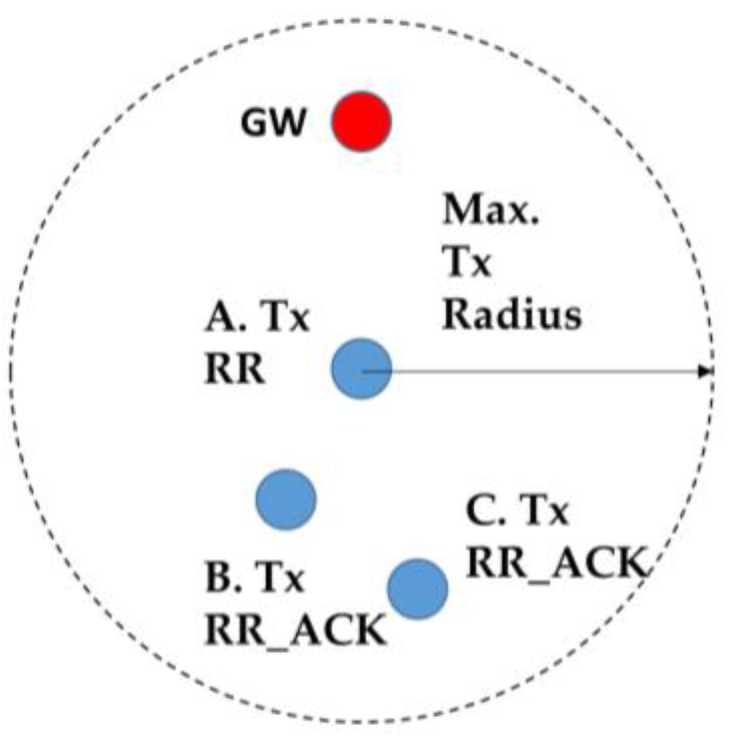
Transmission range for the Route Request (RR) packet.

**Figure 9:**
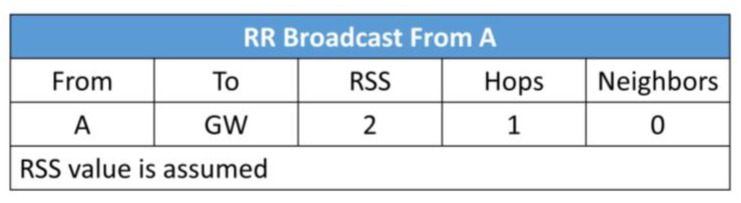
Metrics in the RR packet sent by node A.

**Figure 10:**
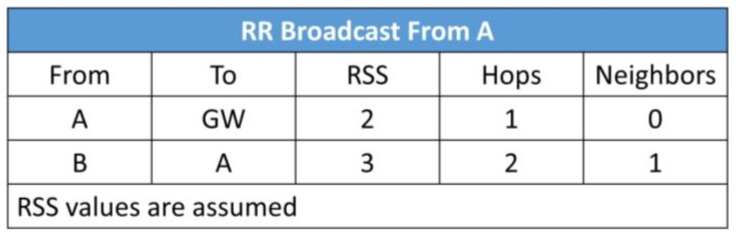
Metrics in the RR packets sent by node B.

1. Suppose node A (Figure 8) broadcasts the RR packets which are received by nodes B and C, because they are in the transmission range of node A;
2. The information contained in the RR packet is shown in Figure 9. The RSS between node A and the gateway (GW) is 2 (assumed). Node A is one hop away from GW and A has no knowledge about its neighbors so far. Node A learns about its neighbors when it broadcasts the RR packet and receives the acknowledgement(s);
3. When nodes B and C send the acknowledgement (RR_ACK), node A learns that it has become part of the routing path of nodes B and C;
4. Now, either B or C broadcasts the RR packet first (depends on the selected time slot);
5. Let us assume that node B will send the RR packet first. When B broadcasts RR packet it contains the information of signal strength between A and itself, number of hops it is away from the gateway through node A, and the number of neighbors (see Figure 10);
6. At this point, C compares the RSS, the least number of hops, and the least number of neighbors to form the routing path with A or B;
7. Node C can choose its path to the gateway by two routes: C → A or C → B → A:
  7.1. If the weight coefficient defined for the least number of hops has a higher value than the other two weight coefficients (for RSS and number of hops), then node C will select node A as the forwarding node for minimum delay in the packet delivery;
  7.2. If the weight coefficient for RSS has a higher value, then C will select B as the forwarding node to save energy.

## 5. Mathematical Analysis

The criteria for path selection are quite straightforward. A node will prefer to form the routing path with the node that has the minimum distance, least number of hops, and least number of neighbors.

The combined values of these parameters are calculated based on the weight given to each parameter. If transmitting power is to be conserved, then signal strength will be given the maximum weight, whereas if the higher throughput is the objective then the least number of hops will be given the maximum weight. Hence, when a node receives the RR packet from multiple nodes n, it stores the signal strength of the received signal (ss), the number of hops between the gateway and the node which sent the RR packet (nh), and the number of neighbors (gh). We propose to use a new score function F(n) evaluated for every node n to make the forwarding node selection. The expression of F(n) is given as follows:

(2) F(n) = (1 + RS(n)) × (1 − HP(n)) × (1 − NG(n)),

where

(3) RS(n )= NS(n)× sswt, {sswt|sswt ∈R, 0≤ sswt≤ 1},

(4) HP(n)= NH(n)× hpwt, {hpwt|hpwt ∈R, 0≤ hpwt≤ 1} ,

(5) NG(n)= NB(n)× ghwt, {ghwt|ghwt ∈R, 0≤ ghwt≤ 1} ,

 sswt, hpwt, and ghwt are the weights for RSS, hops, and neighbors, respectively. The sets of normalized values of RSS, number of hops, and number of neighbors are given by the following expressions, respectively:

(6) $$NS\;=\;\left\{nss_{1},nss_{2},\ldots ,\;nss_{n}\right\},$$

(7) $$NH\;=\;\left\{nhp_{1},nhp_{2},\ldots ,\;nhp_{n}\right\},$$

(8) $$NB\;=\;\left\{ngh_{1},ngh_{2},\ldots \;,\;ngh_{n}\right\}$$

A node i with a score F(i) will be selected as the forwarding node when it fulfills the next rule:

(9) F(i) = max {F(1), F(2), …F(n)}.

Received signal strength (ss) is calculated by:

(10) ss = Txpwr – TL,

where Txpwr is the transmitted signal power in logarithmic units, and TL is the transmission losses, also in dB. The TL parameter is calculated by:

(11) $$TL\;=\;\alpha R\;+\;20log_{10}\left(R\right),$$

where R is the distance between the nodes. The absorption coefficient α (in dB/km) is calculated by [21]:

(12) $$\alpha =\left(\frac{0.11f^{2}}{1+f^{2}}\;+\;\frac{44f^{2}}{4100f^{2}}\right)\;+\;2.75\times 10^{-4}f^{2}\;+\;0.003,$$

where f is the frequency (kHz).

## 6. Packet Header Format

**Figure 11:**
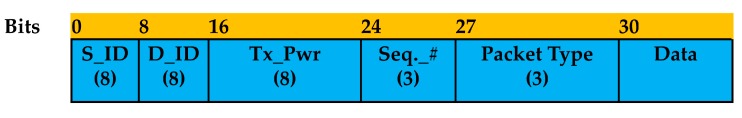
Packet header format.

The header format of the RR, RR_ACK, and RR_RSP packets is shown in Figure 11. There are five header fields, S_ID, D_ID, Tx_Pwr, Seq._#, and Packet Type. The S_ID (source node identification number) and D_ID (destination node identification number) fields have 8 bits length. They are used by the nodes to identify each other. The Tx_Pwr (transmission power) field is used by the receiver node to estimate the path loss. The Seq._# field is used when a packet is transmitted multiple times, e.g., the RR packet is transmitted three times by the gateway. Finally, the receiver node identifies the type of packet received by the packet type field.

## 7. Computer Simulation Results

Simulations were carried out using MATLAB^®^ to estimate two efficiency parameters: the average delay from sending nodes to the gateway, and the average number of hops that each node employed to reach the gateway.

The simulation parameters are given in Table 3.

The average number of hops and delays were simulated for different weight combinations of RSS, number of hops, and number of neighbors. The starting weight was 0.2 and increased to 1 with steps of 0.2. The weight combinations used in the simulation are given in Table 4.

**Figure 12:**
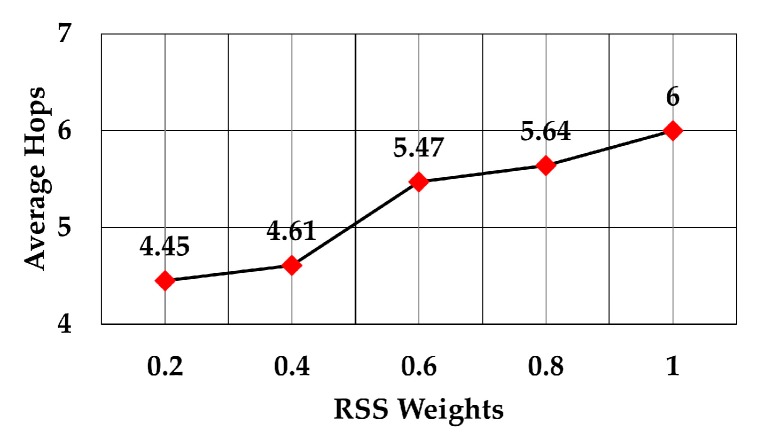
Number of hops against RSS weights.

The average number of hops for RSS weights 0.2 to 1 is shown in Figure 12. The graph shows that the average number of hops increased as RSS weight increased. This means that a node selects the forwarding node which closer to it, although it may have more nodes in the routing path to the gateway. The increase in the number of hops was approximately 34.83% as the RSS weight increased from 0.2 to 1 (6 versus 4.45 average hops).

**Figure 13:**
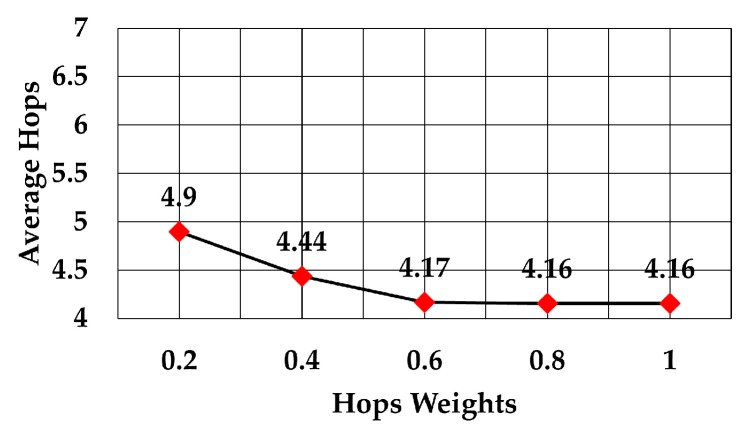
Number of hops against hops weights.

The average number of hops using weights between 0.2 to 1 is shown in Figure 13. The graph shows how that figure decreased as the weight increased. This means that a node prefers to select the forwarding node which needs a smaller number of hops to reach the gateway. In this case, the throughput increased due to the smaller number of hops to reach the gateway, although this may increase the average energy consumption per node. Also, the average number of hops became almost constant after 60% weight of hops. Comparison of the average number of hops to reach the gateway when the RSS weight was the maximum (the other two weights were zero) and when the number of hops weight was the maximum (the other two weights were zero) shows that the average number of hops decreased by more than 30% (6 versus 4.16 average hops).

**Figure 14:**
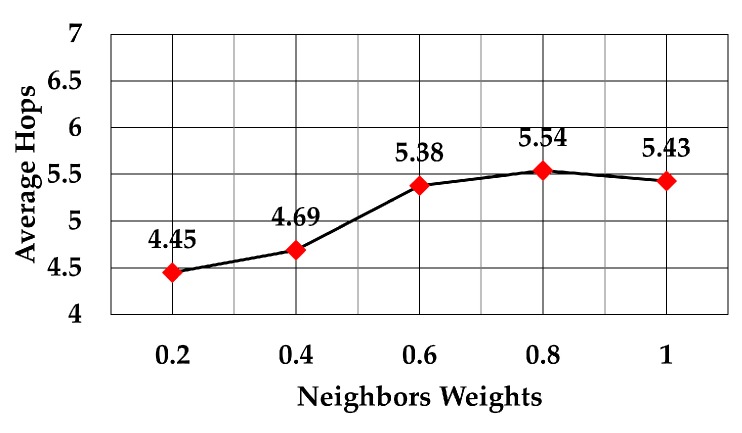
Number of hops against number of neighbors weights.

When the weight for least number of neighbors went from 0.2 to 1, the graph shown in Figure 14 was obtained. Like in case of RSS, the average number of hops increased as the weight for least number of neighbors increased.

**Figure 15:**
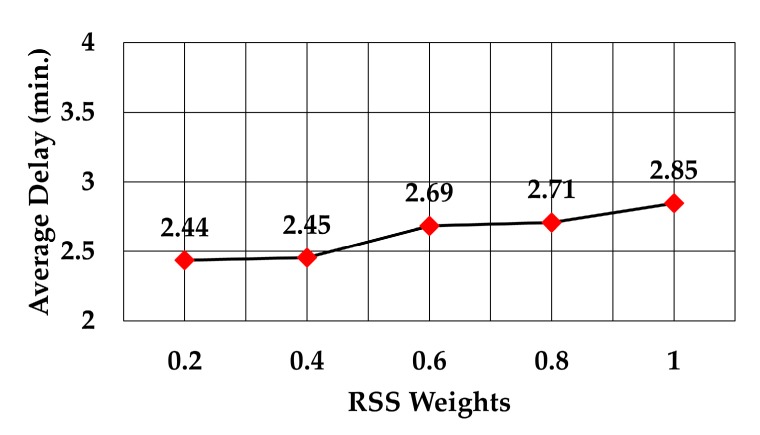
Delay against RSS weights.

**Figure 16:**
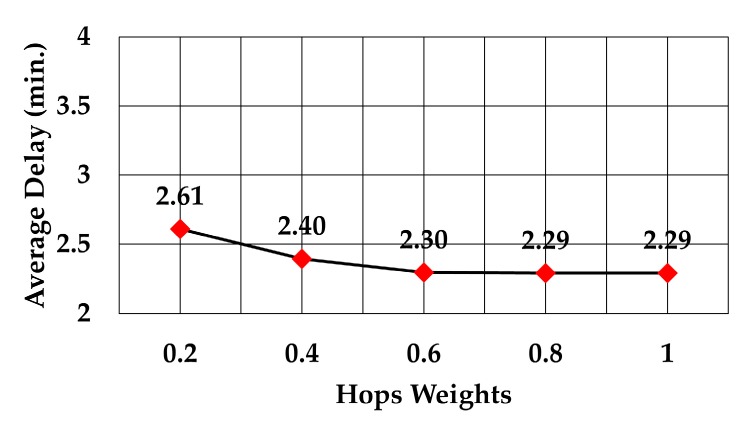
Delay against hops weights.

**Figure 17:**
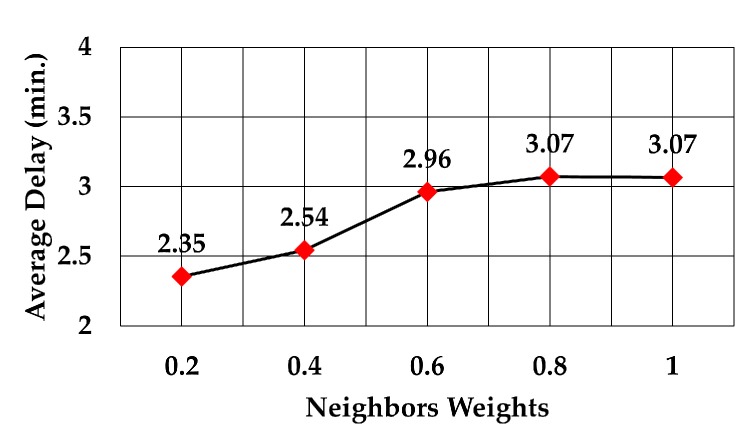
Delay against number of neighbors weights.

The analysis of packet delay from the node sending data to the gateway showed that it followed the same trend as the average number of hops, as can be observed in Figure 15, Figure 16 and Figure 17. In the case of the least number of hops, the maximum weight decreased the delay by 12.2% compared to delay at weight = 0.2 (2.29 versus 2.61 min), 19.6% compared to delay at the maximum weight of RSS (2.29 versus 2.85 min), and 25.4% compared to delay at the maximum weight of least number of neighbors (2.29 versus 3.07 min).

The average energy consumption of a node to send a data packet was analyzed for multiple densities of the nodes. To compare with values given in RECRP [7] we assumed the same simulation parameters. The network area was 10 km × 10 km × 10 km and the number of nodes was 100, 200, 300, 400, 500, and 600. The data packet size was 256 bits and the transmission range was adjusted according to the node’s density. The average energy consumption for multiple transmission range for each number of nodes was simulated as shown in Table 5. The simulation was run 10 times for each transmission range.

**Figure 18:**
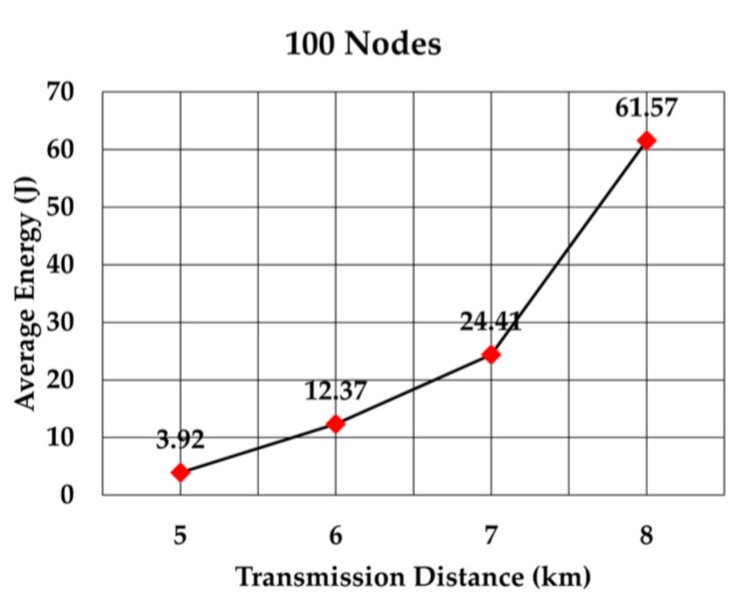
Energy consumption per node per packet for 100 nodes.

**Figure 19:**
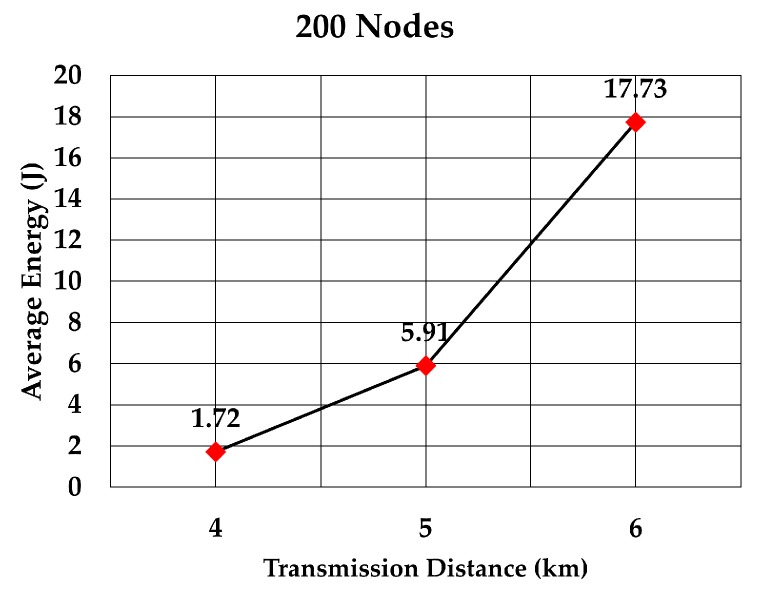
Energy consumption per node per packet for 200 nodes.

**Figure 20:**
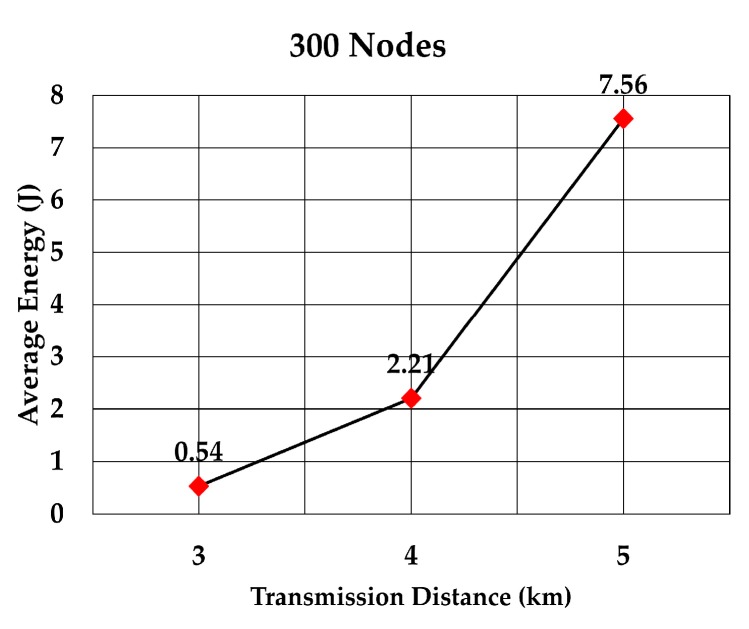
Energy consumption per node per packet for 300 nodes.

**Figure 21:**
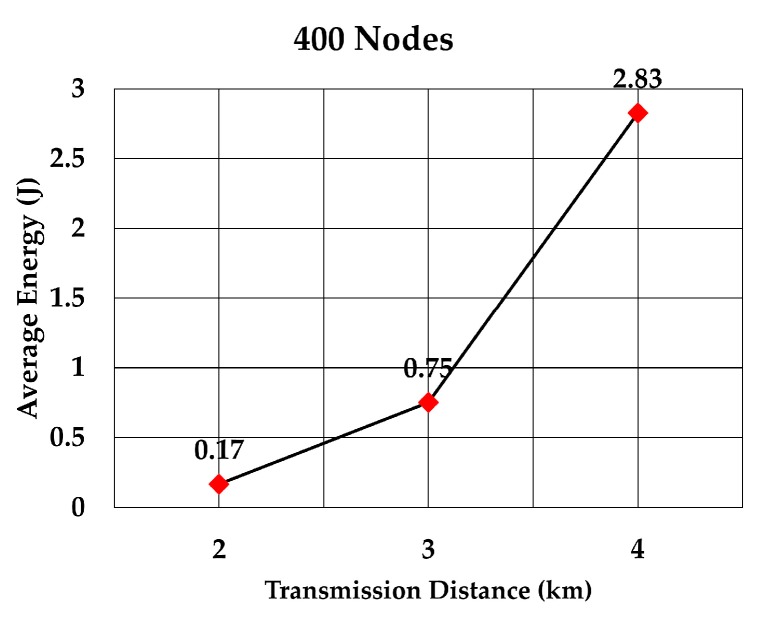
Energy consumption per node per packet for 400 nodes.

**Figure 22:**
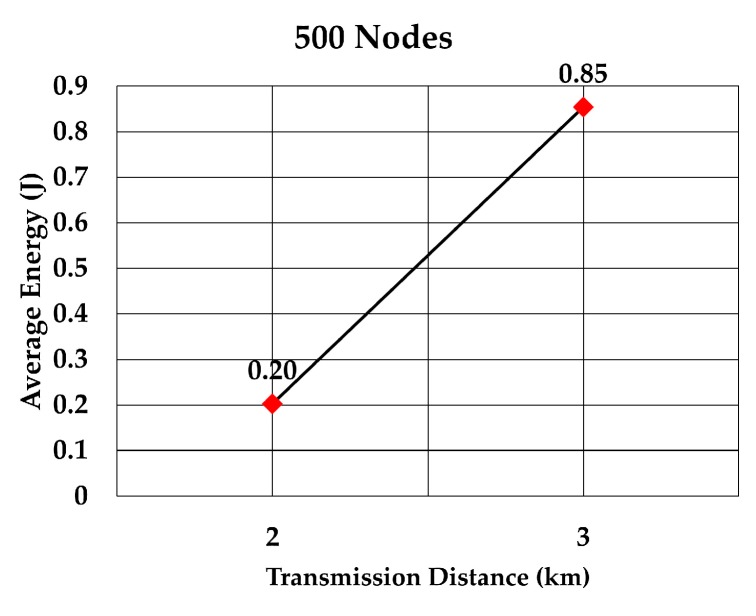
Energy consumption per node per packet for 500 nodes.

**Figure 23:**
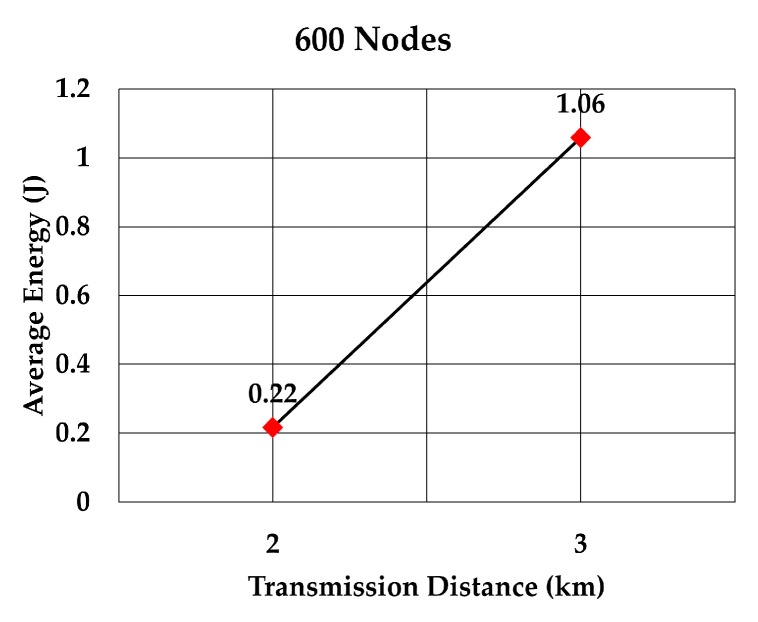
Energy consumption per node per packet for 600 nodes.

The average energy consumption per node per packet for 100, 200, 300, 400, 500, and 600 nodes is shown in Figure 18, Figure 19, Figure 20, Figure 21, Figure 22 and Figure 23, respectively. As we expected, the energy consumption increased as the transmission range increased.

Our proposed protocol is very close to RECRP but there are subtle differences. RECRP is a reactive protocol, whereas our proposed protocol is based on proactive approach. One of the RECRP forwarding nodes selection metrics is the residual energy of the candidate nodes. Instead of residual energy, we considered the number of data packets which a candidate node will have to forward. If a candidate node is already selected as a forwarding node by many neighbor nodes, then it will increase the energy consumption of the forwarding node and decrease the throughput. In order to keep the data traffic balanced among the nodes, the number of neighbors for each node is also a metric for node selection. Our proposed protocol can be maximized in terms of transmission energy by selecting the nodes with the shortest distance. The number of relay nodes between the data sending node and the sink node increases the delay and decreases the throughput. Therefore, our proposed protocol can be maximized to increase the throughput by minimizing the relay nodes. We can also optimize the protocol for energy efficiency by selecting the appropriate weights for distance and number of neighbors, and in the case of throughput, by selecting the appropriate weights for number of hops.

**Figure 24:**
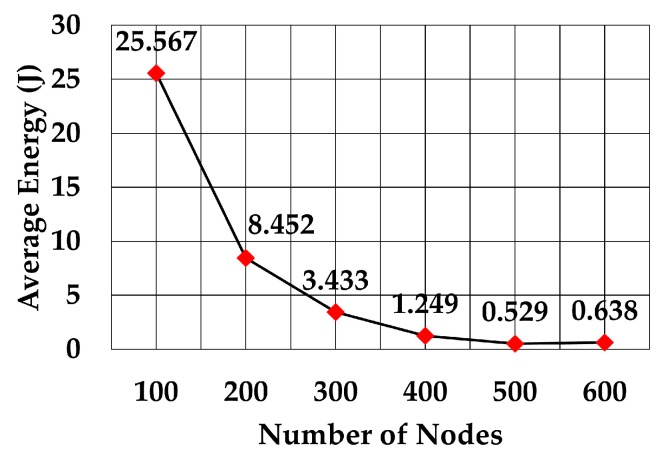
Average energy consumption.

**Figure 25:**
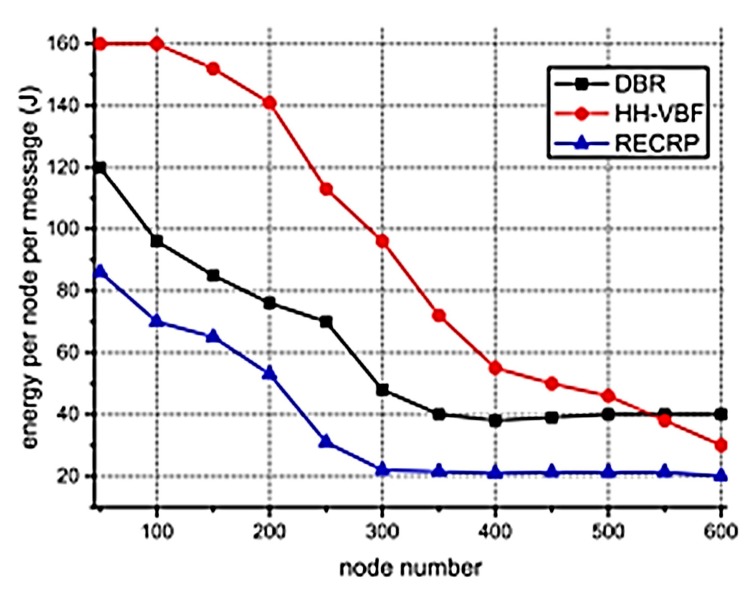
Reliable energy-efficient cross-layer routing protocol (RECRP) energy consumption.

The energy consumption of SPRINT is shown in Figure 24 and energy consumption by RECRP is shown in Figure 25 (taken from [6]). From the comparison of the two graphs, it is obvious that the energy consumption of SPRINT is much lower than RECRP under the same simulation conditions.

The values for RECRP have been tabulated from Figure 23 (approximately) and compared to SPRINT simulations in Table 6.

## 8. Conclusions

In this paper, we presented a routing protocol for randomly deployed underwater network. The sensor nodes are deployed randomly to monitor the environment or to warn of natural disasters, like tsunamis. The protocol is designed to optimize the data throughput and energy consumption of the sensor nodes. This protocol does not require additional sensing devices to ascertain the location. Hence, the proposed routing protocol is based neither on the location of the nodes nor on the topology of the network.

SPRINT is a proactive protocol to minimize the routing delay. Each node that wants to send a packet knows the forwarding node in advance. At regular intervals, when a node receives the data packet, it computes RSS, hops, and neighbors to optimize the routing path and overcome the issue of a dead node. This regular update of the routing path makes the protocol resilient and more efficient. However, computing routing path parameters upon receiving each data packet will increase the energy consumption significantly, therefore the routing path update will be carried out after certain number of data packets, depending on the arrival rate.

Energy consumption is low due to the adaptive transmission power, which is adjusted with the help of RSS estimation. Throughput can also be increased by reducing the relay nodes between the data source node and the gateway. Data traffic among the relay nodes is distributed evenly by considering the number of neighbors at the time of forwarding node selection.

## Figures and Tables

**Table 1:** Packet descriptions.

Packet Name	Description	Code
RR	Route Request	0011001
RR_ACK	Route Request Acknowledgement	1100110
RR_RSP	RR Response	1001010

**Table 2:** List of symbols used in the equations.

Symbol	Description
α	Absorption coefficient (dB/km)
*f*	Frequency (kHz)
ghwt	Number of neighbors weight
HP	Normalized number of hops multiplied by the weight
hpwt	Number of hops weight
NB	Normalized number of neighbors
NG	Normalized number of neighbors multiplied by the weight
NH	Normalized number of hops
NS	Normalized number of RSS
R	Distance between the nodes
RS	Normalized RSS multiplied by the weight
selnode	Selected node
Ss	RSS (dB)
sswt	RSS weight
TL	Transmission loss (dB)
Y	Node selection equation

**Table 3:** Simulation parameters.

Parameter	Value	Unit
Sound speed	1500	m/s
Data rate	5000	bits/s
Frequency	48	kHz
Header size	30	Bits
Transmission power	18	Watts
Nodes	100	nodes
Area	4 × 4	km^2^
Depth	4	km
Nodes density	1.56	nodes/km^3^
Number of runs	100	each

**Table 4:** Weight combinations used in the simulation.

	Weights														
RSS	0.2	0.4	0.6	0.8	1	0.4	0.3	0.2	0.1	0	0.4	0.3	0.2	0.1	0
Hops	0.4	0.3	0.2	0.1	0	0.2	0.4	0.6	0.8	1	0.4	0.3	0.2	0.1	0
Neighbors	0.4	0.3	0.2	0.1	0	0.4	0.3	0.2	0.1	0	0.2	0.4	0.6	0.8	1

**Table 5:** Transmission ranges for different number of nodes.

S. No.	Nodes	Nodes Density	Tx. Ranges (km)
1	100	10	5, 6, 7, 8
2	200	5	4, 5, 6
3	300	3.33	3, 4, 5
4	400	2.5	2, 3, 4
5	500	2	2, 3
6	600	1.66	2, 3

**Table 6:** SPRINT and RECRP energy consumption comparison.

Nodes	Energy Consumption (J)		SPRINT Reduction (%)
	SPRINT	RECRP (Approx.)	
100	25.567	70	63%
200	8.451	53	84%
300	3.432	22	84%
400	1.249	21.5	94%
500	0.528	21	97%
600	0.637	20	96%
